# Oral manifestations of syphilis: A report of six cases

**DOI:** 10.4317/jced.62248

**Published:** 2025-01-01

**Authors:** Manoela Wisniewski Bevervanso, Vanessa Carvajal Soto, Larissa Knysak Ranthum, Helen Heloene Rosa, Eduardo Bauml Campagnoli, Marcelo Carlos Bortoluzzi

**Affiliations:** 1 PhD Student. Universidade Estadual de Ponta Grossa (UEPG)- Campus Uvaranas, Bloco M, Faculdade de Odontologia. Av. General Carlos Cavalcanti, 4748 - Bairro Uvaranas; Ponta Grossa - Paraná, Brasil; 2 MSc Student. Universidade Estadual de Ponta Grossa (UEPG)- Campus Uvaranas, Bloco M, Faculdade de Odontologia. Av. General Carlos Cavalcanti, 4748 - Bairro Uvaranas; Ponta Grossa - Paraná, Brasil; 3 PhD. Universidade Estadual de Ponta Grossa (UEPG)- Campus Uvaranas, Bloco M, Faculdade de Odontologia. Av. General Carlos Cavalcanti, 4748 - Bairro Uvaranas; Ponta Grossa - Paraná, Brasil

## Abstract

Syphilis is a highly contagious infectious disease primarily transmitted through sexual contact, caused by Treponema pallidum, an anaerobic gram-negative bacterium. Oral manifestations are significant, with the oral cavity often being the first site to exhibit symptoms. This study aims to present the clinical manifestations of oral syphilis through a six cases diagnosed in a dental setting. The results reveal that oral syphilis lesions predominantly affect areas such as the tongue, gingiva, and palates, presenting as mucous plaques, ulcerations, and erythematous regions. Painful oral sores and cervical lymphadenopathy were common, with symptoms varying in duration from 3 weeks to 3 months. All cases were classified as secondary syphilis and were confirmed through serological tests.

** Key words:**Syphilis, Treponema pallidum, Oral Cavity, Oral manifestation.

## Introduction

Syphilis is a highly contagious infectious disease primarily classified as a sexually transmitted disease (STD). It is caused by Treponema pallidum, an anaerobic, gram-negative bacterium belonging to the order Spirochaetales. Although the infection is most commonly spread through direct contact with syphilitic lesions during sexual intercourse, it can also be transmitted through non-sexual routes. These include hematological transmission, vertical transmission from mother to child (congenital syphilis), and even direct contact with contaminated objects or lesions. The disease’s primary mode of transmission, however, remains through sexual contact with infected lesions (1-8).

Acquired syphilis progresses through three distinct stages. The primary stage manifests 3 to 90 days post-exposure as a solitary, painless, ulcerated nodule (chancre) that can appear at various inoculation sites, such as the genitals or non-genital areas (ex., oral mucosa, fingers). Secondary syphilis emerges around 3 to 12 weeks after the chancre resolves, though it can occur concurrently. This stage is characterized by diverse muco-cutaneous signs and symptoms including low-grade fever, diffuse lymphadenopathy, headache, myalgia, arthralgia, and pharyngitis. If untreated, primary or secondary syphilis progresses to a latent stage. The latent stage lacks clinical symptoms and can last a lifetime, and in about one third of untreated patients it leads to the tertiary stage. Tertiary syphilis may develop after one year and up to several decades of latency, featuring gummatous disease that causes infiltration and destruction of any organ, with significant effects on the cardiovascular and neurological systems, however it may also occur in oral mucosa and may lead to palatine perforation (1,2,7).

The oral cavity is the main extragenital site of primary syphilis and often the first to exhibit symptoms and in some cases, oral symptoms may be the only indication of the infection. Oral syphilitic manifestations depend upon the stage of the disease and can be multiple and with diverse characteristics (2-6,8,9). This highlights the critical role of dentists in the early recognition, diagnosis, and initiation of necessary therapy.

The primary objective of this case series is to thoroughly document the clinical manifestations of oral syphilis by examining six cases diagnosed in a dental setting, aiming to identify and describe common patterns and variations in the appearance of oral lesions and associated symptoms. As syphilis is a reemerging and potentially fatal disease, albeit prevenTable and curable, this report aims to alert health professionals to the condition and its oral clinical manifestations. 

## Material and Methods

This observational case series study was designed to explore and document the diverse clinical oral manifestations associated with syphilis base on six cases.

Ethical Considerations and Confidentiality: The study received ethical clearance from the Ethical Committee for Human Research at the State University of Ponta Grossa, Brazil, under approval number 6.972.779 (https://www2.uepg.br/propesp-cep/), ensuring adherence to ethical research standards. To safeguard patient confidentiality, all personal identifiers were anonymized before data collection. Each photograph was de-identified prior to analysis by removing any discernible patient information, including facial features or unique identifiers that could potentially reveal the patient’s identity. Oral photographs were taken solely of the intraoral lesions, excluding any unnecessary surrounding anatomical landmarks that might compromise anonymity. All collected data were securely stored, with access limited to the study’s investigators.

Study Design and Population: This research involved a comprehensive analysis of six patients diagnosed with syphilis, observed between 2020 and 2024. Participants included both male and female patients across a broad age range, providing a diverse sample for evaluating the spectrum of oral manifestations associated with syphilis.

Data Collection and Parameters: Clinical data were gathered from each patient. Demographic information, including sex and age, was recorded alongside primary complaints and the duration of symptoms as reported by the patients. Comprehensive clinical descriptions of oral lesions were documented, capturing characteristics such as size, shape, color, and location within the oral cavity. Each lesion was evaluated for associated symptoms.

The presence of lymphadenopathy was assessed, and any associated cutaneous manifestations were noted. Data on comorbidities, such as HIV infection were also collected to understand potential coexisting conditions that might influence the presentation of syphilis.

## Results

This study presents six cases of syphilis diagnosed at the Oral Medicine clinic. All patients in this case series sought consultation primarily due to oral lesions, which were their chief complaint. The duration of lesion development among these patients varied, ranging from 3 weeks to 3 months and the most prevalent symptom reported was painful oral lesions. Among the six patients, five were female, highlighting a notable gender predominance in this case series. The patients’ ages ranged from 18 to 62 years, indicating that oral manifestations of syphilis can occur across a broad age spectrum. Detailed patient information, including demographics, lesion characteristics, and diagnostic results, are comprehensively presented in  Table 1.

**Figure 1:**
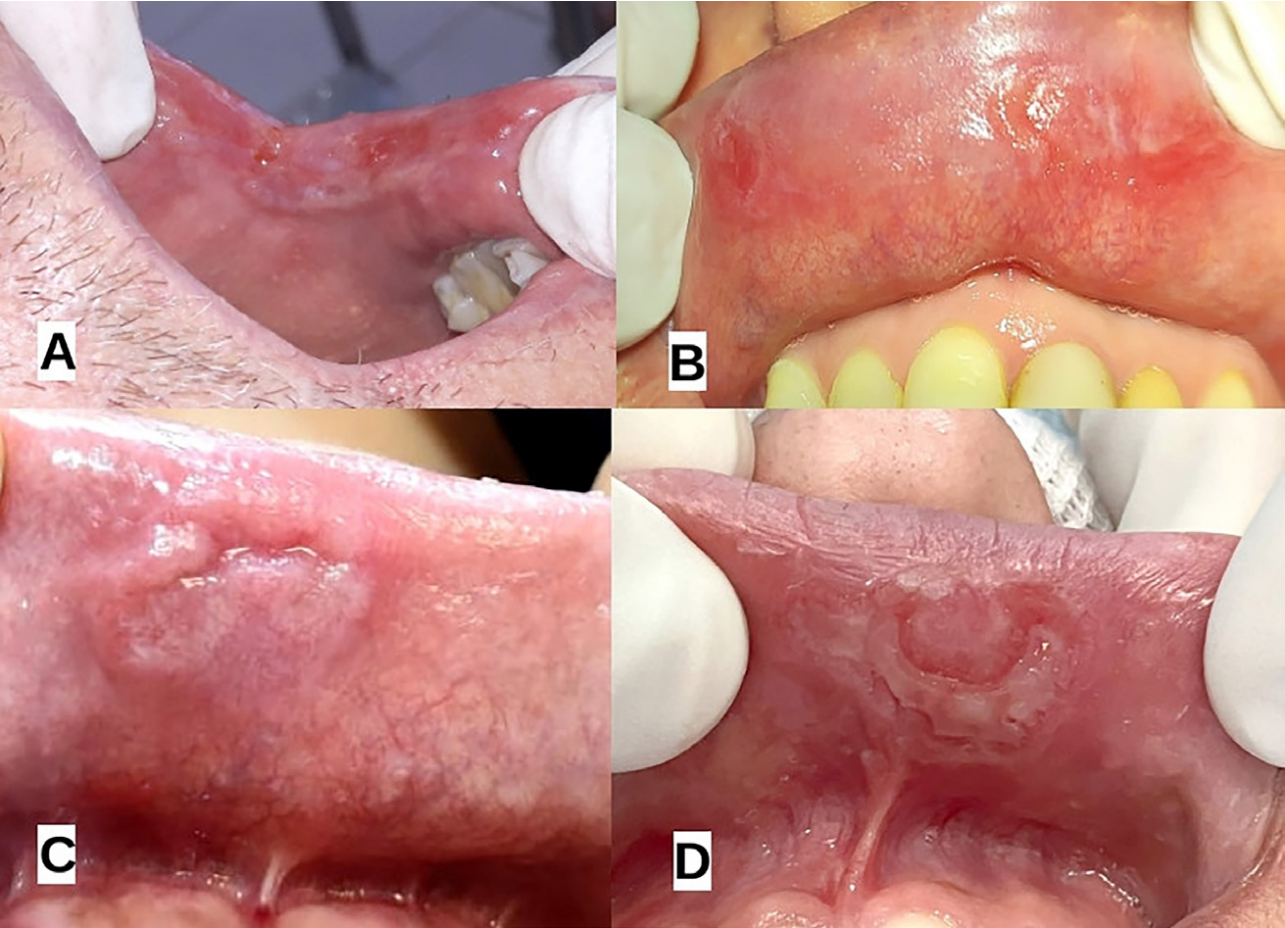
Clinical Presentation of Oral Syphilis on the Lips. This figure showcases the characteristic manifestations of syphilis on the lips, including ulcerative lesions with indurated, raised borders and white striated lesion. A: case 6; B: case1; C: case 4; D: case 5.

**Figure 2:**
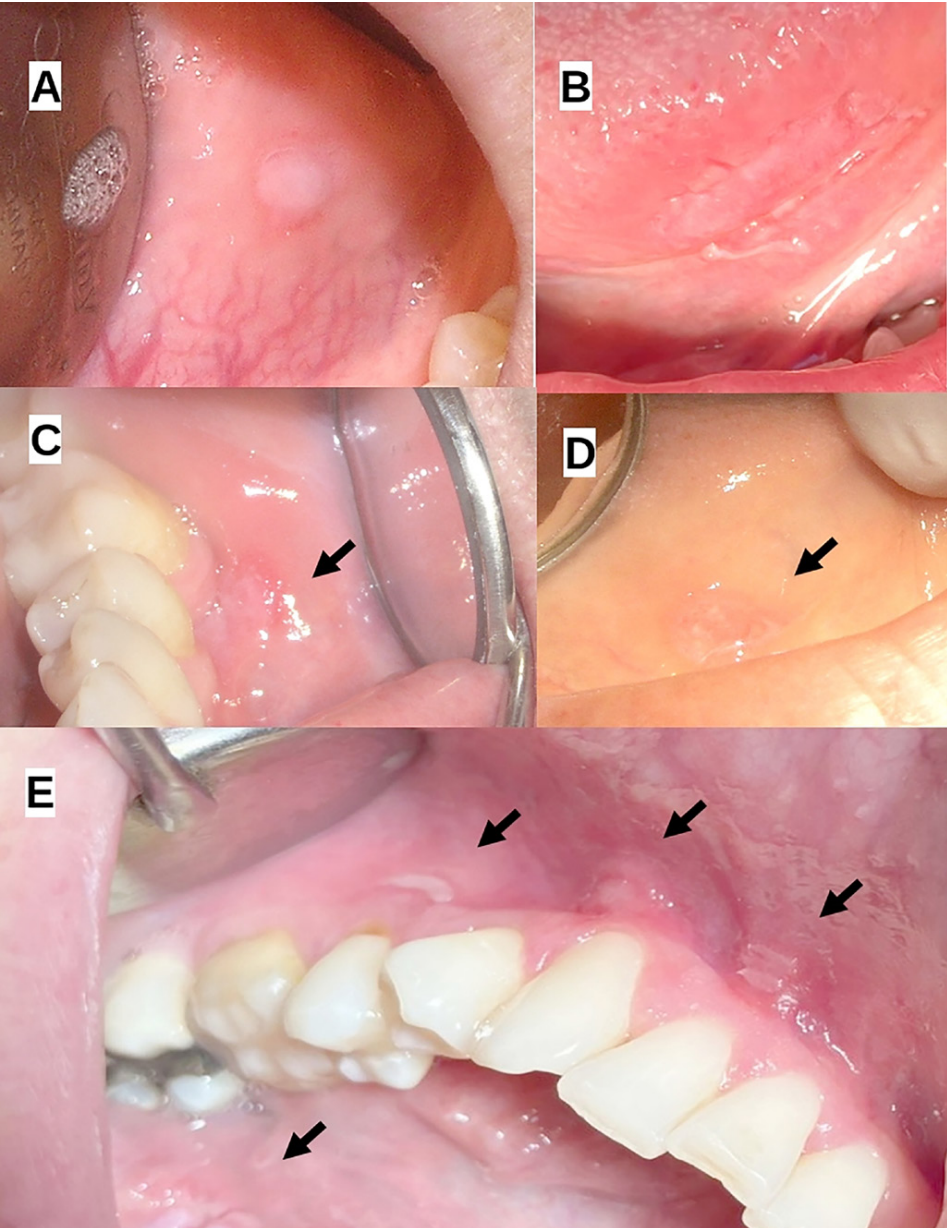
Clinical Presentation of Oral Syphilis on the Tongue and Gingiva. This figure illustrates the typical syphilitic lesions on the tongue and gingiva. Lesions on the tongue appear as whitish, striated mucous plaques surrounded by erythema, while the gingiva exhibits multiple ulcerative lesions with a similar mucous plaque appearance. A: case 2; B: case 3; C: case 1; D: case 1; E: case 3.

**Figure 3:**
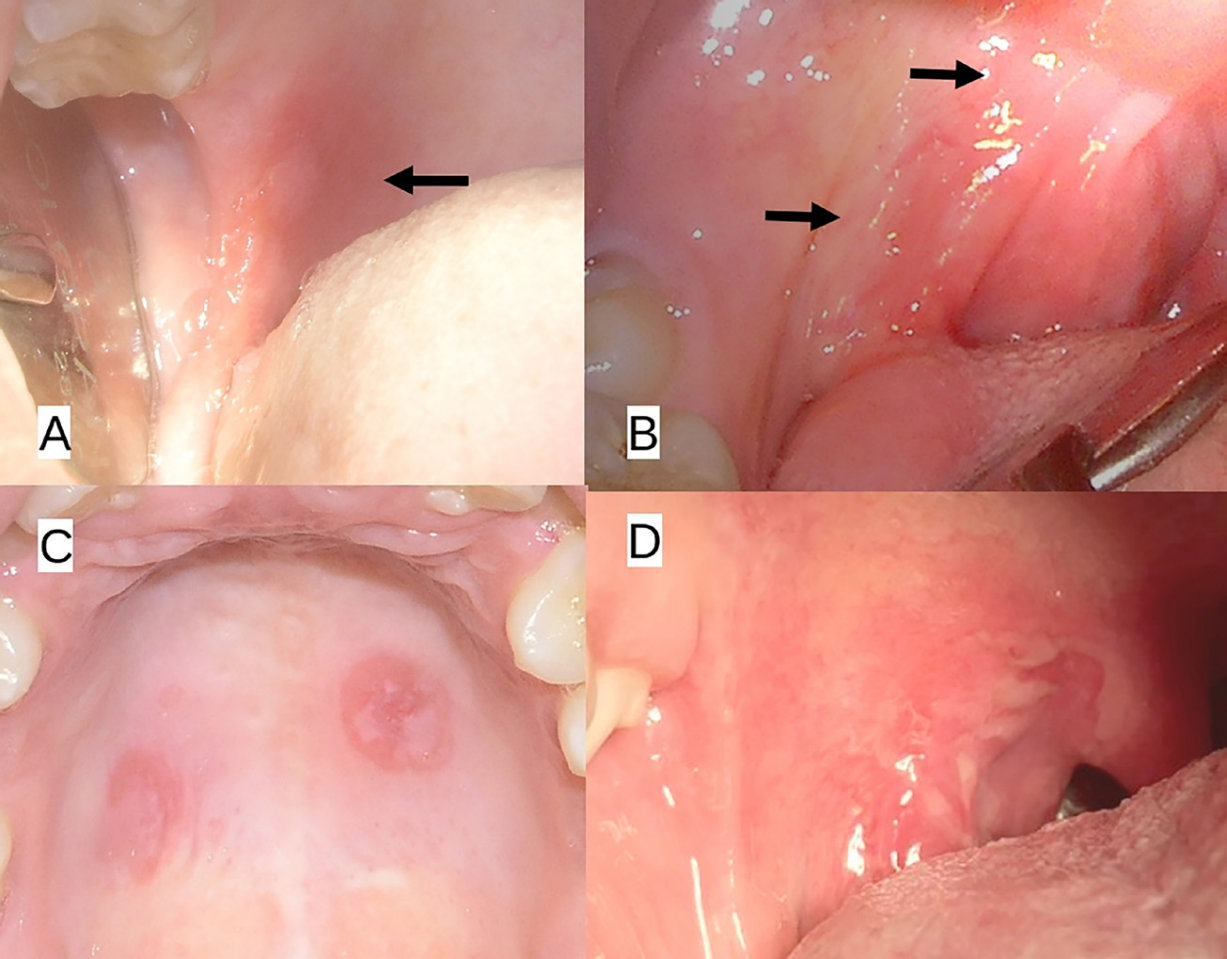
Clinical Presentation of Oral Syphilis on the Palate and Oropharynx. This figure depicts the manifestations of syphilis on the palate and oropharynx, featuring mixed white-erythematous regions and generalized inflammation. Lesions extend to the tonsillar pillars presenting as sore throat. A: case 1; B: case 2; C: case 2; D: case 3.

Oral syphilis lesions were predominantly observed in several key areas of the oral cavity, including the tongue, gingiva, soft and hard palate and lips. The most frequently affected sites included the ventral and lateral aspects of the tongue, where lesions presented as whitish, striated areas and mucous plaques surrounded by erythema. The gingiva and mandibular vestibule featured multiple ulcerative lesions with whitish mucous plaques, while the hard and soft palates exhibited mixed white-erythematous regions and generalized inflammation, often extending to the tonsillar pillars. Lesions on the lips were primarily characterized by ulceration with indurated, elevated borders, and leukoplakia-like appearances. Figure 1 shows the clinical presentation of oral syphilis on the lips, Figure 2 presents the oral characteristics of syphilis on the tongue and gingiva, and Figure 3 presents the oral characteristics of syphilis on the palate and oropharynx.

The predominant clinical features of oral syphilis in these cases included painful oral sores, with many lesions exhibiting a characteristic of mucous plaques, ulceration with raised and irregular borders, and areas of erythema indicative of active inflammation. Cervical lymphadenopathy was observed in 3 patients. The symptoms’ duration ranged from 3 weeks to 3 months, reflecting the variability in the disease’s progression. Additionally, some patients reported more pronounced systemic symptoms such as headaches, weight loss, and nocturnal cough.

Based on the observed period of oral lesion development, progression, and clinical characteristics, all reported cases were classified as secondary syphilis. Each case was confirmed through serological testing, including the Venereal Disease Research Laboratory (VDRL) test, which showed varying titers, and confirmatory tests such as the Fluorescent Treponemal Antibody Absorption (FTA-ABS) and the Microhemagglutination Assay for Treponema pallidum antibodies (MHA-TP). All patients tested negative for HIV, ruling out co-infection that could potentially complicate the clinical presentation and management of syphilis. In one instance, a patient underwent a biopsy prior to the receipt of the VDRL test results, which arrived days later. The diagnosis in this case was subsequently confirmed with specific Treponema pallidum testing ( Table 1).

All patients were referred to the Brazilian public health system for treatment and notification. According to the Clinical Protocol and Therapeutic Guidelines for the Treatment of Syphilis from the Brazilian Ministry of Health, penicillin is the preferred drug for treating syphilis. For primary, secondary, and recent latent syphilis (up to one year in duration), the recommended treatment is a single intramuscular dose of 2.4 million IU of benzathine penicillin G. For late latent syphilis (more than one year in duration) or latent syphilis of unknown duration, as well as tertiary syphilis, the protocol recommends benzathine penicillin G, 2.4 million IU, weekly for three weeks, totaling 7.2 million IU. Alternative treatments include doxycycline and ceftriaxone.

## Discussion

The findings from this study underscore the pivotal role of dentists in identifying and diagnosing syphilis through its oral manifestations. Oral syphilis often presents as the first clinical indication of the disease, particularly in its secondary stage. The lesions observed in this case series exhibited a variety of presentations, including mucous plaques, ulcerations with raised borders, and erythematous regions, primarily affecting the tongue, gingiva, and palates. This variability in presentation highlights the need for dental professionals to maintain a high index of suspicion when encountering atypical oral lesions. Early recognition and diagnosis of syphilis in a dental setting can facilitate appropriate treatment, thereby reducing the risk of disease progression and transmission.

According to the Brazilian Ministry of Health (10), the detection rate of acquired syphilis increased consistently until 2018, after which it stabilized. An exception occurred in 2020, when a decline was observed due to the impact of the COVID-19 pandemic. However, between 2020 and 2021, there was a significant increase in detection rates for acquired syphilis, rising 32.9%, from 59.1 to 78.5 cases per 100,000 inhabitants. Historical data indicates that most reported cases were among males, who accounted for 60.6% of cases. A significant concentration of cases was found in the age groups of 20 to 29 years (35.6%) and 30 to 39 years (22.3%). Notably, among adolescents aged 13 to 19, the incidence of acquired syphilis increased by 2.2 times when comparing the years 2015 and 2021. In 2021, the male-to-female ratio for syphilis cases was 17 men for every 10 women. However, among adolescents, this ratio was reversed, with 7 men for every 10 women affected by syphilis. 

Some patients with undiagnosed syphilis may only present with oral lesions and early recognition of the oral manifestations with appropriated clinical and serological tests will prove imperative (2,3). Syphilis may mimic a variety of other diseases and the oral lesions of the disease is variable and can be nonspecific. These entities include traumatic granulomas and ulcers, pemphigus vulgaris, recurrent aphthous stomatitis and atypical aphthous ulcerations, geographic tongue, fungal infections, tuberculosis, angular cheilitis, squamous cell carcinoma, oral lichen planus, leukoplakia-like lesions among other oral diseases (2,3,5-7,9,11).

The importance of recognizing the oral manifestations of syphilis cannot be understated, as misdiagnosis of this curable disease may allow the evolution of the disease with important consequences for the patients. While syphilis is curable with antibiotics, many cases go undiagnosed for long periods of time and progress to the tertiary stage of the disease may result in potentially fatal neurologic and cardiovascular associated complications (2,3,7).

## Conclusions

This case series highlights the diverse clinical presentations and progression of oral syphilis, emphasizing the importance of early recognition and diagnosis within the dental setting. The study of six cases classified as secondary syphilis, revealed common patterns and variations in oral lesions, ranging from white striated patches to ulcerative plaques, predominantly affecting the tongue, palate, lips, and gingiva. Given the reemergence of syphilis and its potential for severe systemic complications, timely diagnosis and intervention are paramount. This report serves as a reminder of the significant role that dental practitioners play in the early detection and management of this preventable and curable, yet potentially life-threatening disease. By increasing awareness and improving diagnostic acumen, health professionals can contribute to better patient outcomes and help curb the resurgence of syphilis.

## Figures and Tables

**Table 1:** Clinical and Diagnostic Characteristics of Patients with Oral Syphilis Lesions.

Patient	Sex	Age	Presenting Complaint	Evolution time reported	Oral Lesions Locations	Oral Lesions Characteristics	Symptoms Associated with oral lesions	Palpable lymph nodes	Skin Lesions	Other relevant symptoms reported	Blood test
1	F	53	Painful sores on the lip and gums and Pharyngitis (sore throat)	4 weeks	Ventral tongue	White striated lesion resembling lichen planus	Painful	Bilateral cervical	Not observable or reported	Headache	VDRL 1:256 / Confirmed by FTA-ABS/ HIV-Negative
					Mandibular vestibule and gingiva	Whitish mucous plaque surrounded by erythematous or eroded area					
					Soft palate and tonsillar pillar	Whitish mucous plaque surrounded by erythematous area/ generalized inflammation of the pharynx					
					Upper lip	White striated lesion with erythematous and eroded areas					
2	F	18	Painful sores on palate	4 weeks	Lateral tongue	Whitish mucous plaque surrounded by erythematous area	Painful	Bilateral cervical	Palmo-plantar and abdomen	sudden and marked weight loss, nocturnal cough, headache, myalgia	VDRL 1:64 / Confirmed by MHA-TP/ HIV-Negative
					Hard palate	Oval lesions with mixed white-erythematous eroded areas.					
					Soft palate/ tonsillar pillar	slightly whitish area surrounded by erythematous area					
3	F	42	Pain and discomfort in the tongue and throat	3 months	Mandibular vestibule and gingiva	Multiple ulcerative-like lesions and whitish mucous plaque	Pain and discomfort	No	Not observable or reported	No	Confirmed by FTA-ABS/ HIV-Negative
					Soft palate/ tonsillar pillar/ Uvula	Eritematous, ulcerative-like lesions with a covering whitish mucous plaque and membrane					
					Ventral tongue	Whitish fissured mucous plaque					
4	F	34	Painful recurrent mouth ulcers	3 weeks	Lower lip mucosa	Ulceration with indurated and elevated borders with whitish mucous plaque area	Painful	No	Not observable or reported	No	VDRL 1:32 / Confirmed by FTA-ABS/ HIV-Negative
5	F	35	Traumatic injury, thinking it was a tumor	2 months	Upper lip mucosa	Predominantly circular and firm ulceration, with a membrane-covered center	Discomfort	No	Not observable or reported	No	VDRL 1:32 / Confirmed by MHA-TP/ HIV-Negative
6	M	62	Lesion in buccal mucosa	3 months	Lower lip, labial commissure extending to the upper lip	Ulcerated, irregular lesion with raised borders and with areas with whitish center.	Pain on palpation	Unilateral cervical	Genital lesion	No	VDRL 1:32 / Confirmed by FTA-ABS/ HIV-Negative

F= Female/ M= Male. VDRL: Venereal disease research laboratory. FTA-ABS: Fluorescent treponemal antibody absorption. MHA-TP: Microhemagglutination assay for antibodies to T. pallidum.

## Data Availability

The datasets used and/or analyzed during the current study are available from the corresponding author.
